# Characterization of miRNAs associated with Botrytis cinerea infection of tomato leaves

**DOI:** 10.1186/s12870-014-0410-4

**Published:** 2015-01-16

**Authors:** Weibo Jin, Fangli Wu

**Affiliations:** College of Life Science, Zhejiang Sci-Tech University, Hangzhou, Zhejiang 310018 China

**Keywords:** Tomato, High-throughput sequencing, *B. cinerea*-responsive miRNA, Target expression

## Abstract

**Background:**

*Botrytis cinerea* Pers. Fr. is an important pathogen causing stem rot in tomatoes grown indoors for extended periods. MicroRNAs (miRNAs) have been reported as gene expression regulators related to several stress responses and *B. cinerea* infection in tomato. However, the function of miRNAs in the resistance to *B. cinerea* remains unclear.

**Results:**

The miRNA expression patterns in tomato in response to *B. cinerea* stress were investigated by high-throughput sequencing. In total, 143 known miRNAs and seven novel miRNAs were identified and their corresponding expression was detected in mock- and *B. cinerea*-inoculated leaves. Among those, one novel and 57 known miRNAs were differentially expressed in *B. cinerea*-infected leaves, and 8 of these were further confirmed by quantitative reverse-transcription PCR (qRT-PCR). Moreover, five of these eight differentially expressed miRNAs could hit 10 coding sequences (CDSs) via CleaveLand pipeline and psRNAtarget program. In addition, qRT-PCR revealed that four targets were negatively correlated with their corresponding miRNAs (miR319, miR394, and miRn1).

**Conclusion:**

Results of sRNA high-throughput sequencing revealed that the upregulation of miRNAs may be implicated in the mechanism by which tomato respond to *B. cinerea* stress. Analysis of the expression profiles of *B. cinerea-*responsive miRNAs and their targets strongly suggested that miR319, miR394, and miRn1 may be involved in the tomato leaves’ response to *B. cinerea* infection.

**Electronic supplementary material:**

The online version of this article (doi:10.1186/s12870-014-0410-4) contains supplementary material, which is available to authorized users.

## Background

*Botrytis cinerea*, a necrotrophic fungus causing gray mold disease, caused by *Botrytis cinerea* is considered an important pathogen around throughout the world. It induces decay on in a large number of economically important fruits and vegetables during the growing season and during postharvest storage. It is also a majorcreating serious obstacle problem to in long- distance transport and storage [1]. *B. cinerea* infection leads to annual losses of 10 to 100 billion US dollars worldwide [2]. Necrotrophs kill their host cells by secreting toxic compounds or lytic enzymes; they also produce an array of pathogenic factors that can subdue host defenses [3,4]. To limit the spread of pathogens, host cells generate signaling molecules to initiate defense mechanisms in the surrounding cells. Abscisic acid and ethylene are plant hormones that participate in this process [5-7]. Li et al. [8] have found that *SlMKK2* and *SlMKK4* contribute to the resistance to *B. cinerea* in tomato. However, despite extensive research efforts, the biochemical and genetic basis of plant resistance to *B. cinerea* remains poorly understood.

sRNAs are non-coding small RNAs (sRNAs), approximately 21–24 nt in length. These RNAs induce gene silencing by binding to Argonaute (AGO) proteins and directing the RNA-induced silencing complex (RISC) to the genes with complementary sequences. The plant miRNAs are a well-studied class of sRNAs; they are hypersensitive to abiotic or biotic stresses and various physiological processes [9,10]. *miR393* participates in bacterial PAMP-triggered immunity (PTI) by repressing auxin signaling [11]. In *Arabidopsis* plants treated with flg22, *miR393* transcription is induced and the mRNAs of miR393 targets, including three F-box auxin receptors, namely transport inhibitor response 1 (TIR1), auxin signaling F-Box protein 2 (AFB2), and AFB3, are downregulated. Consequently, the resistance to *Pseudomonas syringae,* a bacterial plant pathogen, is increased [11]. miRNAs are also directly involved in the regulation of disease resistance (*R*) genes [12-14]. For example, *nta-miR6019* and *nta-miR6020* are implicated in the regulation of disease resistance in *Nicotiana benthamiana* by controlling the expression of the *N* gene. This gene encodes a Toll and Interleukin-1 Receptor type of nucleotide binding site-leucine-rich-repeat receptor protein that provides resistance to the tobacco mosaic virus [14,15]. The members of different *R*-gene families in tomato, potato, soybean, and *Medicago truncatula* are targeted by miR482 and miR2118 miRNAs [12,13]. In addition, pathogen sRNA can also suppress the host immunity by loading into AGO1 and cause enhanced susceptibility to *B. cinerea* [2].

Tomato (*Solanum lycopersicum*, 2*n* = 24), a widespread member of the *Solanum* species, is an economically important vegetable crop worldwide. Several miRNAs can respond to *B. cinerea* infection in tomato [16]. To investigate the function of miRNAs in the resistance to this pathogen, we constructed two sRNA libraries from mock- and *B. cinerea*-inoculated tomato leaves. These libraries were then sequenced using an Illumina Solexa system. This study was conducted to identify and validate *B. cinerea*-responsive miRNAs from tomato leaves. The outcome of this study could enhance our understanding of the miRNA-mediated regulatory networks that respond to fungal infection in tomato; it could also provide new gene resources to develop resistant breeds.

## Results

### Deep sequencing of sRNAs in tomato

To identify miRNAs that respond to *B. cinerea* infection, two sRNA libraries were constructed from *B. cinerea*-inoculated (TD7d) and mock-inoculated (TC7d) tomato leaves at 7 days post-inoculation (dpi). The libraries were sequenced using an Illumina Solexa analyzer in Beijing Genomics Institute (BGI; China) and the sequences have been deposited in the NCBI Short Read Archive (SRA) with the accession number SRP043615. We generated 33.31 million raw reads from the two sRNA libraries. After removing low-quality tags and adaptor contaminations, we obtained 16,844,708 (representing 6,075,098 unique sequences) and 13,935,908 (representing 4,807,933 unique sequences) clean reads, ranging from 18 nt to 30 nt, from TC7d and TD7d libraries, respectively (Table 1). Most reads (>86% of redundant reads and >77% of unique reads) had at least 1 perfect match with the tomato genome (Table 1).

**Table 1:** **Statistics of the Illumina sequencing of two small RNA libraries including Botrytis cinerea infection and control samples**

**Read data**	TC7d*	TD7d*
Raw reads	18158256	15153960
Reads of appropriate size (18–30 nt)	16844708	13935908
Unique reads of appropriate size	6075098	4807933
Percentage of total reads mapping to S.lycopersicum sl2.40 (100% identity)	87.65%	86.86%
Percentage of unique reads mapping to S.lycopersicum sl2.40 (100% identity)	78.66%	77.61%

*TC7d, Mock-inoculated leaves at 7 dpi; TD7d, *B.cinerea*-inoculated leaves at 7 dpi.

**Figure 1:**
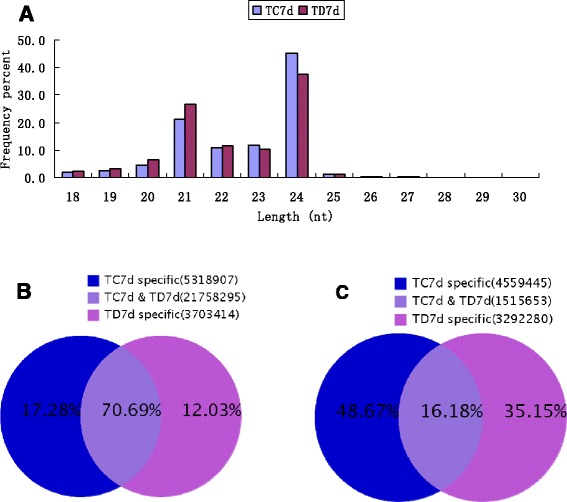
**Size distribution of small RNAs in Mock-inoculated (TC7d) and B.cinerea-inoculated (TD7d) libraries from tomato leaves (A), and Venn diagrams for analysis of total (B) and unique (C) sRNAs between TC7d and TD7d libraries.**

The majority of sRNA reads were from 20 nt- to 24 nt-long. Sequences with 21-nt and 24-nt lengths were dominant in both libraries (Figure 1A). The most abundant sRNAs were 24 nt in length, accounting for 45.15% (TC7d) and 37.65% (TD7d) of the total sequence reads. Our results are consistent with those of previous studies using other plant species such as *Arabidopsis* [17], *Oryza* [18], *Medicago* [19,20], and *Populus* [21]. Moreover, the ratios of the tags differed significantly between the two libraries. The relative abundances of 24-nt sRNAs in the TD7d library were markedly lower than those in the TC7d library; this result suggested that the 24-nt sRNA classes are repressed by *B. cinerea* infection. Nevertheless, the abundance of 21-nt miRNAs was evidently higher in the TD7d library than in the TC7d library, suggesting that the 21-nt miRNA classes are implicated in the response to *B. cinerea* infection. The proportions of common and specific sRNAs in both the libraries were further analyzed. Among the analyzed sRNAs, 70.69% sRNAs common to both libraries; 17.28% and 12.03% were specific to TC7d and TD7d libraries, respectively (Figure 1B). However, opposite results were obtained for unique sRNAs; in particular, the proportions of specific sequences were larger than those of common sequences. Only 16.18% was common to both the libraries; moreover, 48.67% and 35.15% were specific to TC7d and TD7d libraries, respectively (Figure 1C). These results suggested that the expression of unique sRNAs was altered by *B. cinerea* infection.

### Identification of known miRNA families in tomato

Based on unique sRNA sequences mapped to miRBase, release 20.0 [22], with perfect matches and a minimum of 10 read counts, we identified 123 unique sequences belonging to 23 conserved miRNA families in TC7d and TD7d libraries, with total abundances of 90,472 and 137,058 reads per million (RPM), respectively (Table 2). Among the conserved miRNA families, 3 families (miR156, miR166, and miR172) consisted of more than 10 members. In contrast, miR165, miR393, miR394, miR395, and miR477 contained only one member each. Moreover, 20 unique sequences from the 17 non-conserved miRNA families (i.e., conserved only in a few plant species [23]) were detected in TC7d and TD7d libraries. For instance, miR894 has been found only in *Physcomitrella patens* [24]. The majority of non-conserved miRNA families had only one member each; three miRNA families (miR827, miR1919, and miR4376) contained two members (Table 2) each.

**Table 2:** **Known miRNA families and their transcript abundance identified from TC7d and TD7d libraries in tomato**

**conserved miRNA family**	**No. of members**	**miRNA reads count (RPM)**		**Log2 (TD7d/TC7dC)**	**P-value**	**Significance (Up/Down)**
		**TC7d**	**TD7d**			
**Conserved miRNA family**						
miR156	25	39076	85295	1.13	0.0000	** (Up)
miR157	2	481	865	0.85	0.0000	
miR159	2	128	331	1.37	0.00	** (Up)
miR160	2	13	19	0.59	0.0000	
miR162	3	491	527	0.10	0.0000	
miR164	3	100	184	0.88	0.0000	
miR165	1	7	6	−0.07	0.7470	
miR166	19	28611	21493	−0.41	0.0000	
miR167	7	7843	8977	0.19	0.0000	
miR168	7	11938	17420	0.55	0.0000	
miR169	4	4	7	0.71	0.0016	
miR170	2	2	2	0.12	0.7557	
miR171	8	103	83	−0.32	0.0000	
miR172	10	890	772	−0.20	0.0000	
miR319	3	2	8	2.33	0.0000	** (Up)
miR390	4	476	607	0.35	0.0000	
miR393	1	28	30	0.14	0.1483	
miR394	1	1	6	2.23	0.0000	** (Up)
miR395	1	2	3	0.70	0.0585	
miR396	6	147	172	0.23	0.0000	
miR399	5	12	14	0.15	0.2994	
miR477	1	2	2	0.27	0.4504	
miR482	6	115	235	1.03	0.0000	** (Up)
Non-conserved miRNA family						
miR827	2	2	2	0.01	0.9654	
miR894	1	1	1	0.35	0.4469	
miR1446	1	0	2	7.85	0.0000	** (Up)
miR1511	1	1	1	0.91	0.1035	
miR1919	2	86	153	0.83	0.0000	
miR2111	1	1	0	−6.57	0.0001	** (Down)
miR4376	2	180	187	0.06	0.1292	
miR5300	1	515	1401	1.44	0.0000	** (Up)
miR5301	1	54	103	0.93	0.0000	
miR5304	1	7	13	0.81	0.0000	
miR6022	1	975	1317	0.43	0.0000	
miR6023	1	89	101	0.17	0.0015	
miR6024	1	56	103	0.89	0.0000	
miR6026	1	2	2	0.52	0.1671	
miR6027	1	3750	3211	−0.22	0.0000	
miR6300	1	1	3	1.60	0.0002	** (Up)
miR7122	1	1	1	1.07	0.0488	

**Significant difference; Up, Up-regulation; Down, Down-regulation.

**Figure 2:**
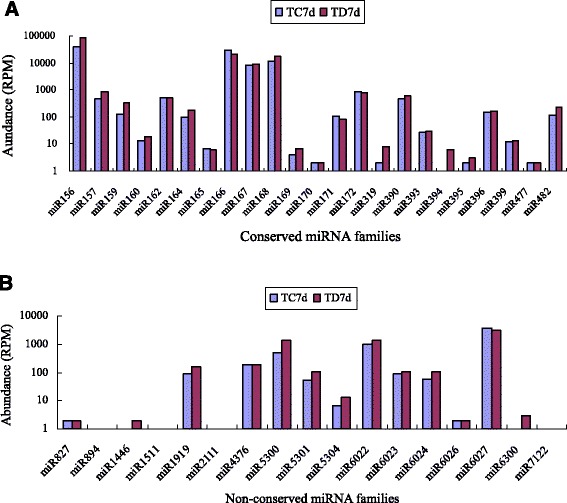
**Reads abundance of conserved miRNA (A) and non-conserved miRNA (B) families in TC7d and TD7d library.**

Read counts differed drastically among the 23 known miRNA families. A few conserved miRNA families such as miR156, miR166, and miR168 showed high expression levels (more than 10,000 RPM) in both the libraries. The most abundantly expressed miRNA family was miR156 with 39,076 (TC7d) and 85,295 (TD7d) RPM, accounting for 43.2% and 62.2% of all the conserved miRNA reads, respectively. miR166 was the second most abundant miRNA family in both the libraries. Several miRNA families, including miR157, miR159, miR162, miR164, miR167, miR171, miR172, miR390, miR396, and miR482, were moderately abundant (Figure 2A). Nevertheless, the most non-conserved miRNA families such as miR827, miR894, and miR1446 showed relatively low expression levels (less than10 RPM) in TC7d and TD7d libraries (Figure 2B). Moreover, different members of the same miRNA family displayed significantly different expression levels (Additional file 1: Table S1). For instance, the abundance of miR156 members varied from 0 to 923,832 reads. These results demonstrated that the expression levels of conserved and non-conserved miRNAs varied dramatically in tomato. The results were consistent with those of previous studies, which showed that non-conserved miRNAs have lower expression levels than conserved miRNAs [25-27].

### Identification of novel miRNA in tomato

**Figure 3:**
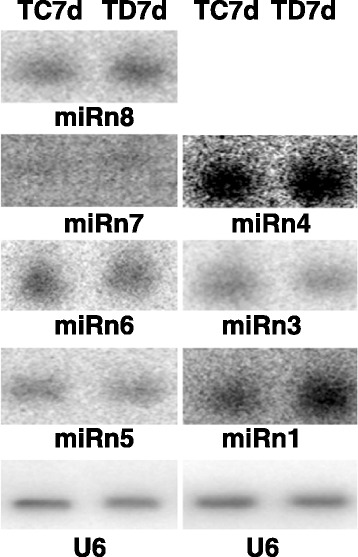
**Validation of novel miRNAs by northern blotting.** RNA gel blots of total RNA isolated from leaves of mock- (TC7d) and *B.cinerea*-inoculated (TD7d) leaves were probed with labeled oligonucleotides. The U6 RNA was used as internal control.

To search for novel miRNAs, we excluded sRNA reads homologous to known miRNAs and other non-coding sRNAs (Rfam 10) and analyzed the secondary structures of the precursors of the remaining 20-nt to 22-nt sRNAs using RNAfold program. The precursors with canonical stem–loop structures were further analyzed using a series of stringent filter strategies to ensure that they satisfied the common criteria established by the research community [28,29]. We obtained 31 miRNA candidates derived from 33 loci, which satisfied the screening criteria. Among those candidates, seven contained miRNA-star (miRNA*) sequences identified from the same libraries; 24 candidates did not contain any identified miRNA* (Additional file 2: Table S2). We considered the seven candidates with miRNA* sequences to be novel tomato miRNAs and the 24 remaining candidates without miRNA* sequences to be potential tomato miRNAs. The secondary structures and sRNA mapping information of the seven novel miRNA precursors are shown in Additional file 3: Figure S1. Gel blot analysis was performed to validate the seven miRNAs and determine their expression patterns. miRn7 had no signal; this was possibly caused by a very low expression in tomato leaves or false-positive results in sRNA sequencing. The six remaining candidates were identified as miRNAs expressed in tomato leaves (Figure 3). In agreement with the sRNA sequencing data, gel blot results showed that miRn1 was upregulated in *B. cinerea*-infected leaves.

**Figure 4:**
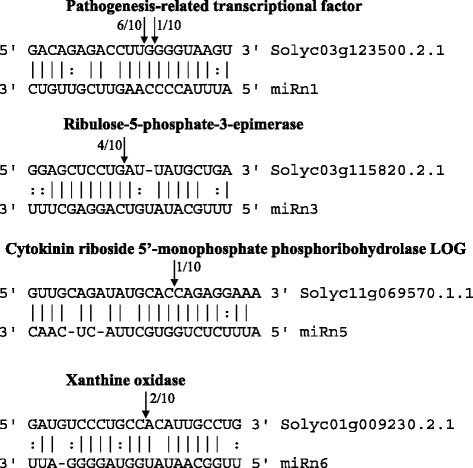
**Cleavage analysis of miRNA targets by 5′ RLM-RACE method.** The identified cleavage sites are indicated by black arrows, and cleavage frequency is presented on top of the arrows.

To validate and functionally identify these six miRNAs, cleaved targets were detected using CleaveLand pipeline. Abundance of the sequences was plotted for each transcript (Additional file 4: Figure S2). We found 26 cDNA targets for five miRNAs (miRn1, miRn3, miRn4-2, miRn5, and miRn6) but none for miRn8. There were 2, 10, 9, and 5 targets in categories 0, 2, 3, and 4, respectively (Table 3). These findings further validated miRn1, miRn3, miRn4-1, miRn5, and miRn6 as novel miRNAs expressed in tomato leaves. miRn1 may target the pathogenesis-related transcriptional factor, indicating that it may be a *B. cinerea*-responsive miRNA. In addition, a total of 10 targets (Solyc03g123500.2.1 and Solyc06g063070.2.1, targeted by miRn1; Solyc03g115820.2.1 and Solyc07g017500.2.1, targeted by miRn3; Solyc04g054480.2.1 and Solyc10g005730.2.1, targeted by miR4-2; Solyc11g069570.1.1 and Solyc12g056800.1.1, targeted by miR5; and Solyc01g009230.2.1 and Solyc06g050650.1.1, targeted by miRn6) were selected for cleavage analysis through 5′ RLM-RACE (5′ RNA ligase mediated rapid amplification of cDNA ends). The results showed that pathogenesis-related transcriptional factor (Solyc03g123500.2.1), Ribulose-5-phosphate-3-epimerase (Solyc03g115820.2.1), Cytokinin riboside 5′-monophosphate phosphoribohydrolase LOG (Solyc11g069570.1.1) and Xanthine oxidase (Solyc01g009230.2.1) were targeted by miRn1, miRn3, miRn5 and miRn6, respectively (Figure 4). The cleavage sites were not found at the expected positions in the seven remaining targets. These results indicated that the four novel miRNAs (miRn1, miRn3, miRn5 and miRn6) would cleave the targets to regulate their expression.

**Table 3:** **Sliced targets were identified using CleaveLand pipline**

**miRNA name**	**Target**	**Cleave site**	**category**	**Target annotation**
miRn1	Solyc03g121180.2.1	816	3	GDSL esterase/lipase At5g22810
miRn1	Solyc03g123500.2.1	370	4	Pathogenesis-related transcriptional factor and ERF, DNA-binding
miRn1	Solyc04g017620.2.1	363	3	Phosphatidylinositol-4-phosphate 5-kinase 9
miRn1	Solyc06g063070.2.1	447	3	Pathogenesis-related transcriptional factor and ERF, DNA-binding
miRn1	Solyc09g008480.2.1	2181	2	Phosphatidylinositol-4-phosphate 5-kinase 9
miRn3	Solyc01g067070.2.1	959	3	Mitochondrial deoxynucleotide carrier
miRn3	Solyc01g111600.2.1	494	3	Metal ion binding protein
miRn3	Solyc03g115820.2.1	1115	2	Ribulose-5-phosphate-3-epimerase
miRn3	Solyc03g118020.2.1	2483	2	RNA-induced silencing complex
miRn3	Solyc06g008110.2.1	1236	2	WD repeat-containing protein
miRn3	Solyc06g074720.2.1	324	4	MKI67 FHA domain-interacting nucleolar phosphoprotein-like
miRn3	Solyc07g017500.2.1	1272	0	Lateral signaling target protein 2 homolog
miRn3	Solyc07g047670.2.1	1347	2	Pescadillo homolog 1
miRn3	Solyc07g066650.2.1	887	3	DCN1-like protein 2, Defective in cullin neddylation
miRn3	Solyc10g076250.1.1	948	2	Aminotransferase like protein
miRn3	Solyc11g006680.1.1	2199	2	Pentatricopeptide repeat-containing protein
miRn4-2	Solyc04g054480.2.1	4328	4	C2 domain-containing protein-like
miRn4-2	Solyc10g005730.2.1	849	4	WD-40 repeat family protein
miRn5	Solyc11g069570.1.1	306	3	Cytokinin riboside 5&apos;-monophosphate phosphoribohydrolase LOG
miRn5	Solyc12g056800.1.1	575	2	Oxidoreductase family protein
miRn6	Solyc01g009230.2.1	4003	2	Xanthine oxidase
miRn6	Solyc02g072130.2.1	1191	3	Protein transport protein SEC61 alpha subunit
miRn6	Solyc05g015680.1.1	144	4	Serine/threonine-protein phosphatase 7 long form
miRn6	Solyc06g050650.1.1	489	3	Serine/threonine-protein phosphatase 7 long form
miRn6	Solyc06g084000.2.1	417	2	Heterogeneous nuclear ribonucleoprotein K
miRn6	Solyc07g042120.1.1	783	0	Serine/threonine-protein phosphatase 7 long form
miR159	Solyc01g009070.2.1	967	0	MYB transcription factor
miR159	Solyc05g053100.2.1	1088	4	Dihydrolipoyl dehydrogenase
miR159	Solyc06g048730.2.1	1010	2	Uroporphyrinogen decarboxylase
miR159	Solyc06g073640.2.1	997	0	MYB transcription factor
miR159	Solyc10g083280.1.1	357	2	evidence_code:10F0H1E1IEG 30S ribosomal protein S.1
miR159	Solyc12g014120.1.1	472	2	evidence_code:10F0H0E1IEG Unknown Protein
miR160	Solyc01g107510.2.1	1843	2	DNA polymerase IV
miR160	Solyc06g075150.2.1	1280	0	Auxin response factor 16
miR160	Solyc09g007810.2.1	1364	4	Auxin response factor 3
miR160	Solyc11g010790.1.1	855	3	Glucosyltransferase
miR160	Solyc11g010800.1.1	447	3	Anthocyanidin 3-O-glucosyltransferase
miR160	Solyc11g010810.1.1	855	4	Glucosyltransferase
miR160	Solyc11g013470.1.1	554	0	Auxin response factor 17 (Fragment)
miR160	Solyc11g069500.1.1	1313	0	Auxin response factor 16
miR169	Solyc01g090420.2.1	1893	2	Armadillo/beta-catenin repeat family protein
miR1919	Solyc03g111340.2.1	1215	4	Ubiquitin-like modifier-activating enzyme 5
miR1919	Solyc12g043020.1.1	1209	3	evidence_code:10F0H1E1IEG Dihydroxy-acid dehydratase
miR319	Solyc06g068010.2.1	702	2	Biotin carboxyl carrier protein of acetyl-CoA carboxylase
miR319	Solyc08g048370.2.1	763	3	Transcription factor CYCLOIDEA (Fragment)
miR319	Solyc08g048390.1.1	1025	2	evidence_code:10F0H1E1IEG Transcription factor CYCLOIDEA (Fragment)
miR394	Solyc01g109400.2.1	488	3	Flavoprotein wrbA
miR394	Solyc01g109660.2.1	298	2	Glycine-rich RNA-binding protein
miR394	Solyc05g015520.2.1	1162	2	F-box family protein
miR394	Solyc06g051750.2.1	1208	2	Cytochrome P450″
miR394	Solyc06g082220.2.1	707	3	Tat specific factor.1
miR394	Solyc12g044860.1.1	1328	2	evidence_code:10F0H1E1IEG ATP dependent RNA helicase
miR5300	Solyc08g068870.2.1	679	2	Aspartic proteinase nepenthesin.1
miR5300	Solyc11g012970.1.1	265	2	Aminoacylase.1

### Identification of *B. cinerea*-responsive miRNAs in tomato

To determine which of the known miRNAs respond to *B. cinerea*, we retrieved the read counts of the 143 unique sequences from 40 known miRNA families from both the libraries; we then normalized these sequences to characterize *B. cinerea*-responsive miRNAs (Additional file 1: Table S1). We identified 57 known miRNAs (from 24 families) that were differentially expressed in response to *B. cinerea* stress (Additional file 5: Table S3). Among these differentially expressed miRNAs, 41 were upregulated and 16 were downregulated in the TD7d library in comparison with the TC7d library. The abundances of 40 miRNA families or the sum of read counts in each miRNA family was calculated and used in differential expression analysis; the results are presented in Table 2. We found that 8 miRNA families were differentially expressed in *B. cinerea*-infected leaves. Seven families, miR159, miR169, miR319, miR394, miR1919, miR1446, and miR5300, were upregulated and only 1 family, miR2111, was downregulated in *B. cinerea*-infected leaves. Thus, the majority of *B. cinerea*-responsive miRNAs or families were upregulated in the TD7d library in comparison with the TC7d library, suggesting that the upregulation of miRNAs is involved in plant responses to *B. cinerea* infection.

### Dynamic expression of *B. cinerea*-responsive miRNA

**Figure 5:**
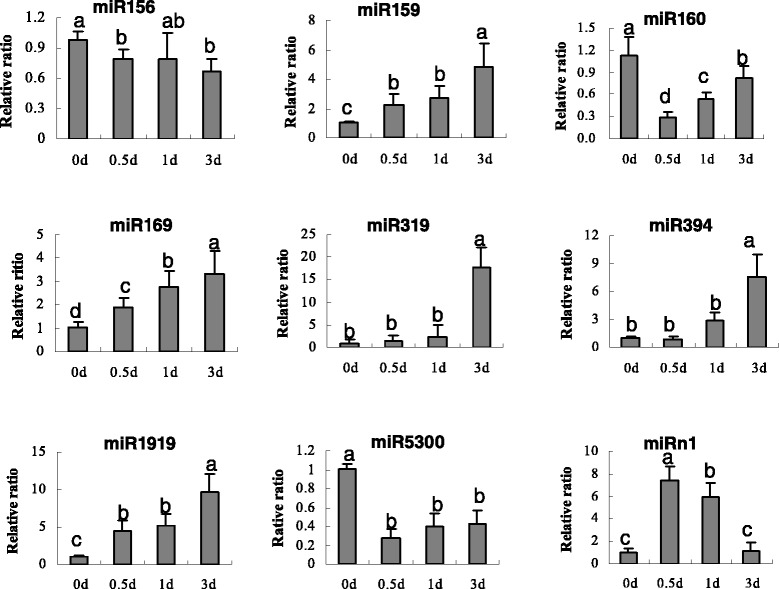
**Quantitative analysis of 9** ***B.cinerea*** **-rsponsive miRNAs by qRT-PCR at 0, 0.5, 1 and 3 day.** U6 RNA was used as the internal control. Error bars indicate SD obtained from three biological repeats.

We also confirmed the Solexa sequencing results and evaluated the dynamic expression patterns of *B. cinerea*-responsive miRNAs at different times after *B. cinerea*-inoculation (0, 0.5, 1, and 3 days). We examined the expression patterns by subjecting 9 *B. cinerea*-responsive miRNAs, including 8 known miRNAs (miR156, miR159, miR160, miR169, miR319, miR394, miR1919, and miR5300) and 1 novel miRNA (miRn1), to quantitative reverse-transcription PCR (qRT-PCR) (Figure 5). The Student’s *t*-test was performed and the probability values of *p* < 0.05 were considered significant. Consistently with sRNA sequencing data, qRT-PCR results showed that 6 miRNAs, miR159, miR169, miR319, miR394, miR1919, and miRn1, were upregulated at each examined time point after *B. cinerea* inoculation. The expression of the first 5 miRNAs increased gradually. In contrast, miRn1 was rapidly upregulated and reached the maximum expression at 0.5 days. miR160 and miR5300, were downregulated; however, no significant differential expression in *B. cinerea*-inoculated leaves was observed for miR156 (Figure 5). These results are consistent with previous data reported by Weiberg et al. [2]. Therefore, these miRNAs, except for miR156, may be involved in the response to *B. cinerea* infection in tomato leaves.

### The expression profiles of the *B. cinerea*-responsive miRNA targets

**Figure 6:**
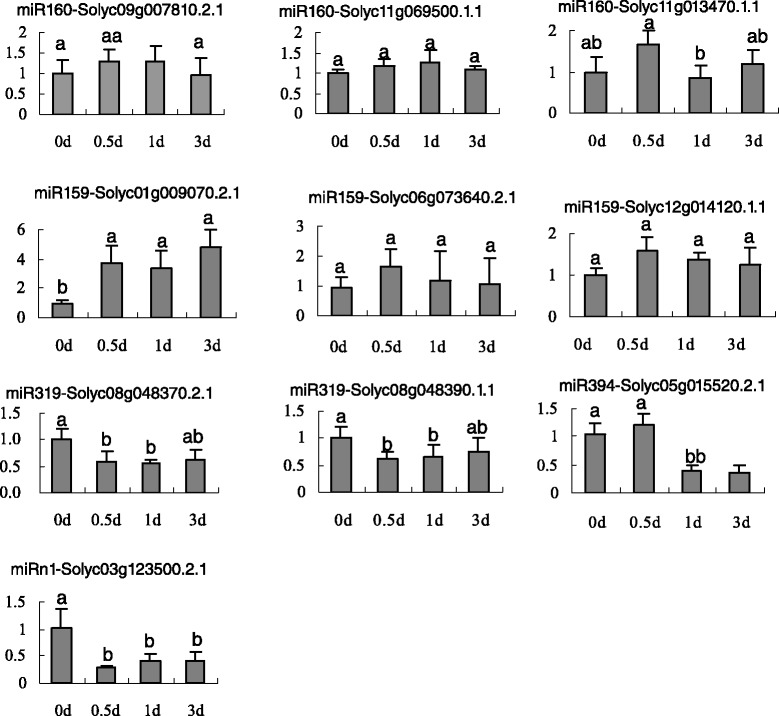
**Quantitative analysis of 10 CDSs targeted by 5** ***B.cinerea*** **-rsponsive miRNAs by qRT-PCR at 0, 0.5, 1 and 3 day.** Actin was used as the internal control. Error bars indicate SD obtained from three biological repeats.

CleaveLand pipeline was performed to predict the targets of the seven known B. cinerea-responsive miRNAs (miR159, miR160, miR169, miR319, miR394, miR1919, and miR5300), thereby detecting the expression profiles of their target genes. The results showed that the seven known miRNAs targeted 28 CDS targets (Table 3). The psRNAtarget program was used for the second screening of the targets, only 9 CDSs were targeted by 4 known miRNAs, namely miR159, miR160, miR319, and miR394 (Additional file 6: Table S4). Moreover, no CDS was predicted as a target of the remaining three miRNAs, namely miR169, miR1919, and miR5300. The expression profiles of these nine target CDSs and Solyc03g123500.2.1 were determined using qRT-PCR at different times (0, 0.5, 1, and 3 d) after the inoculation of *B. cinerea*. The result showed in Figure 6. Two members of the TCP transcriptional factor family (Solyc08g048370.2.1 and Solyc08g048390.1.1), an F-box protein (Solyc05g015520.2.1) and a Pathogenesis-related transcriptional factor (Solyc03g123500.2.1), which were targeted by miR319, miR394 and miRn1, respectively, were significantly downregulated in *B. cinerea*-inoculated leaves at different times (Figure 6), and exhibited a negative relationship to the expression of the 3 miRNAs (Figure 5). However, a MYB transcriptional factor (Solyc01g009070.2.1), which was targeted by miR159, was significantly upregulated and exhibited a consistent expression pattern with that of miR159. In addition, no significant differential expression in *B. cinerea*-inoculated leaves was observed in the remaining five target CDSs (Figure 6). Therefore, the results strongly suggested that the miR319, miR394 and miRn1 may be involved in the responses to *B. cinerea* infection in tomato leaves.

## Discussion

miRNAs have been found as post-transcriptional regulators in many eukaryotic plants and are involved in the response to various environmental stresses [30,31]. To identify tomato miRNAs associated with the resistance to *B. cinerea*, we performed high-throughput sequencing of TD7d and TC7d libraries constructed from *B. cinerea*- and mock-inoculated tomato leaves, respectively. The results showed substantially higher abundance of 21-nt miRNAs in the TD7d library than in the TC7d library, indicating that the upregulation of the 21-nt miRNA classes may be important in the response to *B. cinerea* infection. The relative abundances of 24-nt sRNAs in the TD7d library were markedly lower than those in the TC7d library. Plant 24-nt small interfering RNAs (siRNAs) are mostly derived from repeats and transposons. These 24-nt siRNAs trigger DNA methylation at all CG, CHG, and CHH (where H = A, T, or C) sites, resulting in H3K9me2 modifications [32]. These modifications reinforce transcriptional silencing of transposons and genes that harbor or are adjacent to repeats or transposons in *Arabidopsis* [33-38]. In this study, the decreased number of 24-nt sRNAs in TD7d library suggested that the levels of DNA methylation at some specific loci are reduced in response to *B. cinerea* infection. We could reasonably assume that the reduced DNA methylation exposes some host genes, which could enhance the resistance or susceptibility to *B. cinerea* infection. Further research will be necessary to prove these assumptions.

**Figure 7:**
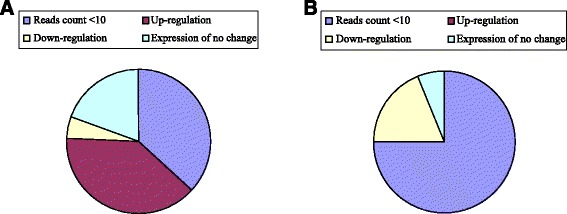
**Match analysis for the 57 miRNA profiles in this study and previous reported data** **[** 2 **].** The Match analysis for 41 miRNAs **A)** and 16 miRNAs **B)** which were up- and down-regulated in the TD7d library in comparison with the TC7d library, respectively.

In this study, 57 known miRNAs from 24 families were differentially expressed in the response to *B. cinerea* stress (Additional file 5: Table S3). Among these differentially expressed miRNAs, 41 were upregulated and 16 were downregulated in the TD7d library compared with those in the TC7d library. We compared the expression profiles of these 57 differentially expressed miRNAs with the published data on *B. cinerea*-infected tomato leaves at 0, 24, and 72 h after inoculation [2]. A total of 27 miRNAs presented low read counts (<10) in the three libraries (Figure 7). The total read count in each of TC7d and TD7d was approximately two to the three times higher than that in the three libraries. Most of the 27 miRNAs presented lower read counts than the 20 miRNAs in the present study. Among the remaining 30 miRNAs, most differentially expressed miRNAs also showed consistent expression profiles between our data and the reported data (Figure 7).

We obtained 31 novel miRNA candidates derived from 33 loci, which satisfied the screening criteria. Seven of these novel miRNA candidates contained miRNA* sequences identified from the same libraries, whereas 24 candidates did not contain any identified miRNA* sequences (Additional file 2: Table S2). We performed a gel blot analysis to validate these seven novel miRNAs and determine their expression patterns. MiRn7 was not expressed, but miRn6 was expressed in mock- and *B. cinerea*-infected leaves (Figure 3). This finding is inconsistent with the sRNA-seq data, in which miRn7 exhibited higher read count than miRn6 (Additional file 2: Table S2). We speculated that few miRNAs may show inconsistent abundance values when examined using two different methods, i.e., Northern blot and sRNA-seq.

*miR319* is a conserved miRNA that mediates the changes in plant morphology [39-43]. Some microarray data suggest that this miRNA is also involved in plant responses to drought and salinity stress; transgenic plants of creeping bentgrass (*Agrostis stolonifera*) with an overexpressed rice *miR319* gene have enhanced resistance to drought and salt stress [44]. Our results showed that transient overexpression of *miR319* may increase the resistance of tomato plants to *B. cinerea*.

*miR394* is a conserved miRNA found in several plant species [45-48]. Liu et al. [49] have found that high salinity upregulates the expression of *miR394* in *Arabidopsis*. The expression of *miR394b* in roots and *miR394a* and *miR394b* in shoots is initially upregulated and then downregulated under iron-deficient conditions [50]. In *Brassica napus*, *miR394a, b,* and *c* are upregulated in the roots and stems under sulfate-deficient conditions [47]. Similarly, the expression of *miR394a, b,* and *c* in all plant tissues is induced by cadmium treatment [47]. Song et al. [51] have reported that *miR394* and its target, the F-box gene *At1g27340*, are involved in the regulation of leaf curling-related morphology of *Arabidopsis*. The available data suggest that *miR394* is involved in the development and abiotic stress regulation. Furthermore, transgenic plants overexpressing the *Arabidopsis miR319a* gene may have enhanced drought resistance but diminished salt tolerance [52]. In this study, we found that the transient overexpression of *miR394* may also increase the resistance of tomato leaves to *B. cinerea*.

## Conclusions

This study was the first to perform a genome-wide identification of miRNAs involved in resistance against *B. cinerea* by using sRNA sequencing and transient overexpression in tomato leaves. We identified 174 miRNAs, including 143 known and 31 novel miRNAs, by using the high-throughput sequencing data of *B. cinerea*-infected and mock-infected tomato leaves. Among these 174 miRNAs, 58 were differentially expressed in *B. cinerea*-stressed leaves. Our study showed that the upregulated miRNAs may play important roles in the response to *B. cinerea* infection in tomato plants. We also found that that upregulated miRNAs inhibited the expression of their targets. Hence, these miRNAs may be involved in the response to *B. cinerea* infection in tomato leaves.

## Methods

### Plants, *B. cinerea* inoculation, and RNA extraction

Tomatoes (*S. lycopersicum*) cv. Jinpeng 1 were used as host plants; they were grown in a greenhouse at a 16-h day/8-h night cycle, at 22–28°C. At the age of 6 weeks, plants were inoculated using a solution containing *B. cinerea* conidia (2 × 10^6^ spores ml^−1^), 5 mM glucose, and 2.5 mM KH_2_PO_4_. The inoculation solution was applied to both leaf surfaces using a soft brush. After inoculation, the plants were kept at 100% relative humidity to ensure spore germination. The *B. cinerea*- and mock-inoculated leaves were harvested at 5 time points (0 days, 0.5 days, 1 days, 3 days, and 7 days) after treatment, in 3 biological replicates. We found that the *B. cinerea* spores appeared on the leaves at 7 dpi. The 7-dpi leaves of *B. cinerea*-infected (TD7d) and control (TC7d) plants were sent to BGI (Shenzheng, China) for the deep sequencing of sRNAs. The samples were frozen in liquid nitrogen and stored at −70°C for the studies of transcript expression.

Total RNAs were extracted from leaf tissues using TRIzol reagent (Invitrogen, Carlsbad, CA, USA), followed by RNase-free DNase treatment (Takara, Dalian, China). Their concentrations were quantified using a NanoDrop ND-1000 spectrophotometer.

### Identification of novel miRNAs in tomato

For the prediction of novel miRNAs, the unique sequences with a minimum raw reads count of 10 in each library were extracted and combined into 1 sRNA library for miRNA prediction; all reads that matched to tomato coding RNA, tRNA, rRNA, or known miRNA sequences with 2 mismatches were removed. The remaining reads were mapped to genomic sequences from ftp://ftp.solgenomics.net/tomato_genome/wgs/assembly/build_2.40 using Bowtie with a maximum of 2 mismatches [53]. With 1 end anchored 20 bp away from the mapped sRNA location, sequences of 120 to 360 bp with each extension of 20 bp that covered the sRNA region were collected. Secondary structures of each sequence were predicted using the RNAfold tool from the Vienna package (version 1.8.2) [54]. Under conditions similar to those suggested by Meyers et al. [28] and Thakur et al. [29], stem–loop structures with ≤3 gaps involving ≤8 bases at the sRNA location and miRNA–miRNA* duplexes accounting for more than 75% reads mapping to the precursor locus were considered candidate miRNA precursors. Finally, the candidate miRNAs matching with no mismatch to all plant miRNAs deposited into miRBase database (Version 20.0) [22] were considered to be conserved miRNAs and the remaining were considered to be novel miRNA candidates.

### Identification of *B. cinerea*-responsive miRNAs

The frequency of miRNAs from the 2 libraries was normalized to 1 million by total clean reads of miRNAs in each sample (RPM). If the normalized read count of a given miRNA was zero, the expression value was modified to 0.01 for further analysis. The fold-change between the TD7d and TC7d libraries was calculated using following the equation: Fold-change = log_2_ (TD7d/TC7d). The miRNAs with fold-changes of >2 or <0.5 and *p*-values of ≤0.001 were considered to be upregulated or downregulated in response to *B. cinerea* stress, respectively. The *p*-value was calculated according to the previously established methods [55].

### Validation of identified miRNAs using RNA gel blot

For each sample, a 100 μg-aliquot of RNA was resolved on a 15% polyacrylamide/1× TBE/8 M urea gel and subsequently transferred to a GeneScreen membrane (NIN). DNA oligonucleotides that were perfectly complementary to candidate miRNAs (Additional file 7: Table S5) were end-labeled with [γ-^32^P]ATP using T4 polynucleotide kinase (New England Biolabs) to generate highly specific probes. Hybridization and washing procedures were performed as described previously [9]. The membranes were briefly air-dried and then read in a phosphoimager.

### Identification of miRNA targets

For identifying the miRNA targets, the degradome data of tomato leaves was downloaded from NCBI GEO database (accession number: GSM553688). The FASTA files of tomato CDS sequences were downloaded from the ftp site ftp://ftp.solgenomics.net/genomes/Solanum_lycopersicum/nnotation/ITAG2.3_release/ITAG2.3_cds.fasta. Following this, CleaveLand pipeline was first employed for detecting the cleaved targets of miRNAs [56,57]. The online psRNAtarget program was further used for target identification (http://plantgrn.noble.org/psRNATarget/?function = 3).

### Target validation of RLM-RACE analysis

miRNA-mediated target gene cleaveage was confirmed using total RNA by 5′ RLM-RACE, as previously described [58]. In brief, poly (A) + RNA was isolated from cucumber leaves using a magnetic mRNA isolation kit (NEB, UK). The cleaved products were uncapped and carried a free phosphate, thereby allowing direct ligation with the RNA adaptor RA44 using T4 RNA Ligase (Ambion, USA). The ligation products were extracted using phenol/chloroform and precipitated with glycogen before first-strand cDNA synthesis was performed using SuperScript II Reverse Transcriptase (Invitrogen, USA). Nested PCR was performed using *premix ExTaq*™ Hot Start Version (TaKaRa, Dalian, China) and RA44OP/IP and GSP1/GSP2 primers in order to detect the cleaved products. The amplicons were further confirmed by sequencing. The adaptor and primers used for 5′ RLM-RACE analysis are listed in Additional file 7: Table S5.

### Quantitative real-time PCR analysis

Expression profiles of the *B. cinerea*-responsive miRNAs were assayed by qRT-PCR. Total RNA was treated with RNase-free DNase I (TaKaRa, Dalian, China) to remove genomic DNA. Forward primers for 5 selected miRNAs were designed based on the sequence of the miRNAs and are listed in Additional file 7: Table S5. The reverse transcription reaction was performed with the One Step PrimeScript miRNA cDNA Synthesis Kit (TaKaRa, Dalian, China) according to the manufacturer’s protocol [20].

SYBR Green PCR was performed following the manufacturer’s instructions (Takara, Japan). In brief, 2 μl of cDNA template was added to 12.5 μl of 2× SYBR Green PCR master mix (Takara), 1 μM each primer, and ddH_2_O to a final volume of 25 μl. The reactions were amplified for 10 s at 95°C, followed by 40 cycles of 95°C for 10 s and 60°C for 30 s. All reactions were performed in triplicate, and the controls (no template and no RT) were included for each gene. The threshold cycle (C_T_) values were automatically determined by the instrument. The fold-changes for miR811 and miR845 were calculated using 2^−ΔΔCt^ method, where ΔΔC_T_ = (C_T,target_ − C_T,inner_)_Infection_ − (C_T,target_ − C_T,inner_)_Mock_ [59].

### Availability of supporting data

The data sets supporting the results of this article are included within the article and its additional files. The sRNA-seq data sets of TC7d and TD7d libraries are available in NCBI SRA database under accession number SRP043615. The clean reads of TC7d and TD7d data sets are also available in Additional files 8 and 9.

## Additional files

Additional file 1: Table S1. Identification and characterization of known miRNA members. Additional file 2: Table S2. Identification and expression analysis of novel miRNAs in tomato. Additional file 3: Figure S1. Identification of the novel miRNAs. Additional file 4: Figure S2. Target plots (t-plots) of miRNAs targets confirmed by using degradome sequencing in tomato. Additional file 5: Table S3. The differential expression of known miRNAs. Additional file 6: Table S4. Prediction the targets of *B.cinerea*-responsive miRNAs via *psRNAtarget*. Additional file 7: Table S5. Primers used in this study. Additional file 8:
**TC7d.tar.gz.** Compressed file of TC7d sRNA-seq data with a minimum raw reads count of 2. Additional file 9:
**TD7d.rar.gz.** Compressed file of TD7d sRNA-seq data with a minimum raw reads count of 2.

## Abbreviations

qRT-PCR: quantitative reverse-transcription PCR
CDS: Coding sequence
miRNAs: microRNAs
*B.cinerea*: *Botrytis cinerea*
sRNAs: small RNAs
AGO: Argonaute
RISC: RNA-induced silencing complex
PTI: PAMP-triggered immunity
TIR1: Transport inhibitor response 1
AFB2: Auxin signaling F-Box protein 2
dpi: Days post inoculation
RPM: Reads per million
miRNA*: miRNA-star
siRNA: small interfering RNAs
RLM-RACE: RNA ligase mediated rapid amplification of cDNA ends
