# Supplementary material for: Characterization of miRNAs associated with Botrytis cinerea infection of tomato leaves
Source: BMC Plant Biol. 2015 Jan 16;15:1. doi: 10.1186/s12870-014-0410-4 (PMC4311480; doi:10.1186/s12870-014-0410-4)
Supplement: Additional file 3: Figure S1. — Identification of the novel miRNAs. [file 12870_2014_410_MOESM3_ESM.pdf]

```
>miRn1
mature sequence     ATTTACCCCAAGTTCGTTGTC
processing frequency    0.89
obs                                          ffffffSSSSSSSSSSSSSSSSSSSSSlllllllllllllllll//lllllllllllllllllllllllllllMMMMMMMMMMMMMMMMMMMMMffffff
pri_seq                                      guucuacaacguacguaggguaaguggauggcugaaauucguua//auugaucaucauggauuuaagccauccauuuaccccaaguucguugucaaacgc
pri_struct                                   (((..((((((.((...((((((((((((((((.((((((((..//.)))))....)))))))).))))))))))))))))...)).))))))..)))..        #MM
t08607804_x13                                .....acaacguacguaggguaagugga................//......................................................        0
t04896233_x34                                ......caacguacguaggguaag....................//......................................................        0
t08973963_x30                                ......caacguacguaggguaagu...................//......................................................        0
t01355201_x21                                ......caacguacguaggguaagug..................//......................................................        0
t05605324_x43                                ......caacguacguaggguaagugg.................//......................................................        0
t03832112_x12                                .......aacguacguaggguaagugga................//......................................................        0
t02346705_x15                                ............................................//...........................auuuaccccaaguucguug........        0
t07165488_x1256                              ............................................//...........................auuuaccccaaguucguuguc......        0
t01845419_x35                                ............................................//............................uuuaccccaaguucguuguca.....        0

--   cu      U  GUA                g        ggaaauacuucucugcaggacaauagccuagccguggcccu  -    ----       ----        a-  a   gaaag   ga
  guu  aCAACG AC   GGGUAAGUGGauggcu aaauucgu                                         gc uuca    uuuccau    uaccggau  uc aca     guu  a
  |||  |||||| ||   |||||||||||||||| ||||||||                                         || ||||    |||||||    ||||||||  || |||     |||
  caa  UGUUGC UG   CCCAUUUAccuaccga uuuaggua                                         cg aagu    aaaggug    gugguuua  ag ugu     cag  a
cg   aC      U  AAC                a        cuacuaguuagaagcuauucaaa------------------  u    uuag       ugag        gg  a   aacaa   aa


>miRn3
mature sequence     TTTGCATATGTCAGGAGCTTT
processing frequency    0.44
obs                                          fffffffSSSSSSSSSSSSSSSSSSSSSllllllllllllllllllllllllllllllllllllllllllllllMMMMMMMMMMMMMMMMMMMMMfffffffffffffffffffffff
pri_seq                                      uagauugagcugcugaccuauggauuccucagcccuaucuauuuaugauuucaaacauauagauagguuugugguuugcauaugucaggagcuuuauuuuacccuuuguuugaucauu
pri_struct                                   .((((.(((((.(((((.((((.(..((((((.((((((((..((((........)))))))))))).))).)))..).)))).))))).))))).)))).................      #MM
t03977250_x85                                .......agcugcugaccuauggau.............................................................................................      0
t03093666_x325                               .......agcugcugaccuauggauu............................................................................................      0
t05250426_x227                               .......agcugcugaccuauggauuc...........................................................................................      0
t03028432_x370                               .......agcugcugaccuauggauucc..........................................................................................      0
t09162263_x52                                .......agcugcugaccuauggauuccu.........................................................................................      0
t00274470_x14                                .........................................................................guuugcauaugucaggag...........................      0
t03284876_x10                                .........................................................................guuugcauaugucaggagcuu........................      0
t04500317_x480                               .........................................................................guuugcauaugucaggagcuuu.......................      0
t02633262_x49                                .........................................................................guuugcauaugucaggagcuuua......................      0
t00393630_x50                                .........................................................................guuugcauaugucaggagcuuuau.....................      0
t04614024_x14                                ..........................................................................uuugcauaugucaggagcuu........................      0
t04719452_x867                               ..........................................................................uuugcauaugucaggagcuuu.......................      0
t00094227_x245                               ..........................................................................uuugcauaugucaggagcuuua......................      0
t03905480_x30                                ..........................................................................uuugcauaugucaggagcuuuau.....................      0
t02281299_x10                                ...........................................................................uugcauaugucaggagcuuu.......................      0
t01275524_x12                                ...........................................................................uugcauaugucaggagcuuuau.....................      0

------------------u    u     G     C    G UU   -   c        uu    auu
                   agau gAGCU CUGAC UAUG A  CCu cag ccuaucua  uaug   u
                   |||| ||||| ||||| |||| |  ||| ||| ||||||||  ||||
                   uuua UUCGA GACUG AUAC U  ggg guu ggauagau  auac   a
uuacuaguuuguuucccau    U     G     U    G UU   u   u        --    aaa


>miRn4-1
mature sequence     TTGGCTGAGTGAGCATCACGG
processing frequency    0.99
obs                                          fffffffffffffffffffffffffffffffffSSSSSSSSSSSSSSSSSSSSSllllllllllllllllllllllllllllllllllllllllllllllllllllllllllllllllllMMMMMMMMMMMMMMMMMMMMMfffffffffffffff
pri_seq                                      cggauuugggguucugagauucaguugauuucucaggugcucacucagcuaauaguuauuauuuaagaaacucaauaauauuggcagcaaggagaauggugacuuucaggaugauaacuauuggcugagugagcaucacggaaaucgacgugauuc
pri_struct                                   ((((........)))).((.(((((((((((((..(((((((((((((((((((((((((((((..((((.(((......(((....)))........))).))))).)))))))))))))))))))))))))))))..))))))))))).))).))        #MM
t03984575_x55                                .................................caggugcucacucagcuaaua......................................................................................................        0
t01185842_x14                                ....................................................................................................................acuauuggcugagugagcauc...................        0
t07503333_x99                                .......................................................................................................................auuggcugagugagcaucacgg...............        0
t04378577_x125                               ........................................................................................................................uuggcugagugagcauca..................        0
t01869422_x404                               ........................................................................................................................uuggcugagugagcaucac.................        0
t02709315_x201                               ........................................................................................................................uuggcugagugagcaucacg................        0
t04097381_x161090                            ........................................................................................................................uuggcugagugagcaucacgg...............        0
t07136142_x653                               ........................................................................................................................uuggcugagugagcaucacgga..............        0
t08965390_x39                                ........................................................................................................................uuggcugagugagcaucacggaa.............        0
t01904439_x414                               .........................................................................................................................uggcugagugagcaucacgg...............        0
t00556349_x90                                ..........................................................................................................................ggcugagugagcaucacgg...............        0
t08913056_x12                                .............................................................................................................................ugagugagcaucacggaaauc..........        0
t04991482_x37                                .................................................................................................................................ugagcaucacggaaaucgacg......        0

cggauuugggguucuga  u   -          CA                             aa    c   auaaua---   g
                 ga uca guugauuucu  GGUGCUCACUCAGCUAAUAguuauuauuu  gaaa uca         uug c
                 || ||| ||||||||||  |||||||||||||||||||||||||||||  |||| |||         |||
                 cu agu cagcuaaaGG  CUACGAGUGAGUCGGUUaucaauaguagg  cuuu agu         aac c
-----------------  u   g          CA                             a-    c   aagaggggu   g


>miRn4-2
mature sequence     TTGGCTGAGTGAGCATCACTG
processing frequency    0.72
obs                                          fffffffffffffffffffffffffffffffffSSSSSSSSSSSSSSSSSSSSSllllllllllllllllllllllllllllllllllllllllllllllllllllllllllllllllllMMMMMMMMMMMMMMMMMMMMMffffffffffffffff
pri_seq                                      cugauuugggguuuugagauucaguugauuucugaggugcucacucagcuaauaguuauuguuuaagaaacucaauaauauuggcagcaaggagaauggugacuuucaggaugauaacuauuggcugagugagcaucacugaaaucgacaugauucu
pri_struct                                   ................(((.((((((((((((.(.(((((((((((((((((((((((((((((..((((.(((......(((....)))........))).))))).))))))))))))))))))))))))))))).).))))))))).))).)))       #MM
t03088162_x58                                .................................gaggugcucacucagcuaaua.......................................................................................................       0
t01185842_x14                                ....................................................................................................................acuauuggcugagugagcauc....................       0
t05059083_x14                                .......................................................................................................................auuggcugagugagcaucacu.................       0
t00420936_x31                                .......................................................................................................................auuggcugagugagcaucacug................       0
t04378577_x125                               ........................................................................................................................uuggcugagugagcauca...................       0
t01869422_x404                               ........................................................................................................................uuggcugagugagcaucac..................       0
t00412121_x2776                              ........................................................................................................................uuggcugagugagcaucacu.................       0
t07480569_x9096                              ........................................................................................................................uuggcugagugagcaucacug................       0
t00561348_x24                                ........................................................................................................................uuggcugagugagcaucacuga...............       0
t01150231_x20                                .........................................................................................................................uggcugagugagcaucacu.................       0
```

```
t02913903_x24                                .........................................................................................................................uggcugagugagcaucacug................      0
t05452254_x10                                ..........................................................................................................................ggcugagugagcaucacug................      0
t02303790_x64                                .................................................................................................................................ugagcaucacugaaaucgaca.......      0

cugauuugggguuuug   u   -         u A                             aa    c   auaaua---   g
                aga uca guugauuuc G GGUGCUCACUCAGCUAAUAguuauuguuu  gaaa uca         uug c
                ||| ||| ||||||||| | |||||||||||||||||||||||||||||  |||| |||         |||
                ucu agu cagcuaaaG C CUACGAGUGAGUCGGUUaucaauaguagg  cuuu agu         aac c
----------------   u   a         U A                             a-    c   aagaggggu   g


>miRn5
mature sequence     ATTTCTCTGGTGCTTACTCAAC
processing frequency    0.79
obs                                          ffffffMMMMMMMMMMMMMMMMMMMMMMllllllllllllllllllllllllllllllllllllllllllllllllllllllllllllllllllllllllllllSSSSSSSSSSSSSSSSSSSSSfffff
pri_seq                                      uaguugauuucucuggugcuuacucaacuaauaguuauuauuuaagaaaaucaauaauauuggaagcaaggagaauggagacuaucaggaugauaacuauugguugagcagcauuagagaaauugacaug
pri_struct                                   ..((((((((((((((((((..(((((((((((((((((((((.......((.......(((....)))........))........))))))))))))))))))))).))))))))))))))))))...  #MM
t05483602_x235                               ......auuucucuggugcuuacucaac......................................................................................................  0
t08395337_x76                                .......uuucucuggugcuuacucaac......................................................................................................  0
t09258282_x49                                ........................................................................................................ugagcagcauuagagaaauug.....  0

-ua                  AU                     aagaaaa-  aauaaua-   g
   guugAUUUCUCUGGUGCU  CUCAACuaauaguuauuauuu        uc        uug a
   ||||||||||||||||||  |||||||||||||||||||||        ||        |||
   caGUUAAAGAGAUUACGA  GAGUugguuaucaauaguagg        ag        aac c
gua                  C-                     cuaucaga  uaagaggg   g


>miRn6
mature sequence     TTGGCAATATGGTAGGGGATT
processing frequency    0.71
obs                                          ffffffSSSSSSSSSSSSSSSSSSSSSllllllllllllllllllllllllllllllllllllllllllMMMMMMMMMMMMMMMMMMMMMffffff
pri_seq                                      uuccaauucccuaccauauugccaaauauuugccuuuaucuuuguguucaucuagcuaaagacaaauguuuggcaauaugguaggggauucugguc
pri_struct                                   ..((((((((((((((((((((((((((((((.(((((.((.((....))...)).))))).)))))))))))))))))))))))))))..)))..    #MM
t00437381_x18                                ......uucccuaccauauugccaaau.....................................................................    0
t02757911_x31                                .....................................................................uuggcaauaugguaggggauu......    0
t09205397_x20                                ......................................................................uggcaauaugguaggggauuc.....    0

uu   --                           c     u  u--  u
  cca  aUUCCCUACCAUAUUGCCAAAUauuug cuuua cu   ug g
  |||  ||||||||||||||||||||||||||| ||||| ||   ||
  ggu  UAGGGGAUGGUAUAACGGUUuguaaac gaaau ga   ac c
cu   cU                           a     c  uuc  u


>miRn7
mature sequence     TGAGCAATGTAGGTCTAGACT
processing frequency    0.95
obs                                          fffffSSSSSSSSSSSSSSSSSSSSSlllllllllllllllllllllllllllllllllllllllllllllllllllllllllllllllllllllllllllllllllllllllllllllllllMMMMMMMMMMMMMMMMMMMMMfffff
pri_seq                                      gcuagucuagaccuacguugcucggacuccaaaaauuguugcagcaccacuaucagauccuccaaaaugcacuauuucugaaggauccggcacccagccaucgacaauuuuaaagagucugagcaauguaggucuagacuaaggu
pri_struct                                   ..(((((((((((((((((((((((((((..((((((((((..............((((((.(((((((...))))).)).)))))).(((.....)))..))))))))))...)))))))))))))))))))))))))))....   #MM
t02630108_x22                                .....ucuagaccuacguugcucgga.......................................................................................................................   0
t06356850_x11                                .......................................................................................................................ugagcaauguaggucuagac......   0
t07649812_x200                               .......................................................................................................................ugagcaauguaggucuagacu.....   0

--gc                           ca-          cagcaccacuauca      c  -
    uagUCUAGACCUACGUUGCUCGGAcuc   aaaauuguug              gauccu ca aaaugc
    |||||||||||||||||||||||||||   ||||||||||              |||||| || ||||| a
    aUCAGAUCUGGAUGUAACGAGUcugag   uuuuaacagc              cuagga gu uuuaua
ugga                           aaa          uaccgacccacggc      a  c


>miRn8
mature sequence     TCTGTGACAGGATTATGGAAG
processing frequency    0.41
obs                                          fffffSSSSSSSSSSSSSSSSSSSSSSSSlllllllllllllllllllllllllllllllllllllllllllllllllllllllllllllllllllllllllMMMMMMMMMMMMMMMMMMMMMfffffffffffffffffffffffffff
pri_seq                                      acaucuuccgguaguccugucgcagaugacucucgaucuugucauuaagccccuuucucuaagucuaauuaauuugaauucaaugugguaggacgagagucaucugugacaggauuauggaagaucgaguuaucaaagggcguuuucuuc
pri_struct                                   ..((((((((.((((((((((((((((((((((((.(((((((((.........(((..................)))......))))))))))))))))))))))))))))))))))))))))))..........((((......)))).      #MM
t05771110_x162                               .....uuccgguaguccugucgcagaug..........................................................................................................................      0
t00342234_x116                               .....uuccgguaguccugucgcagauga.........................................................................................................................      0
t08253593_x32                                ......uccgguaguccugucgcagaug..........................................................................................................................      0
t00655972_x110                               ............................................................................................acgagagucaucugugacagg.....................................      0
t08286250_x24                                ............................................................................................acgagagucaucugugacagga....................................      0
t06944474_x18                                .............................................................................................cgagagucaucugugacagga....................................      0
t03534100_x36                                .....................................................................................................aucugugacaggauuauggaaga..........................      0
t01862847_x99                                ......................................................................................................ucugugacaggauuaugg..............................      0
t08661411_x40                                ......................................................................................................ucugugacaggauuauggaa............................      0
t04282538_x349                               ......................................................................................................ucugugacaggauuauggaag...........................      0
t07553568_x126                               ......................................................................................................ucugugacaggauuauggaaga..........................      0
t05694952_x109                               ......................................................................................................ucugugacaggauuauggaagau.........................      0

-----------------------ac        G                        a         gccccuuaa   ucuaaguc
                         aucuUCCG UAGUCCUGUCGCAGAUGacucucg ucuugucau         uuc        u
                         |||||||| |||||||||||||||||||||||| |||||||||         |||
                         uaGAAGGU AUUAGGACAGUGUCUacugagagc aggauggug         aag        u
cuucuuuugcgggaaacuauugagc        -                        -         uaacuu---   uuuaauua
```
