# Supplementary figures and images for: Characterization of miRNAs associated with Botrytis cinerea infection of tomato leaves
Source: BMC Plant Biol. 2015 Jan 16;15:1. doi: 10.1186/s12870-014-0410-4 (PMC4311480; doi:10.1186/s12870-014-0410-4)

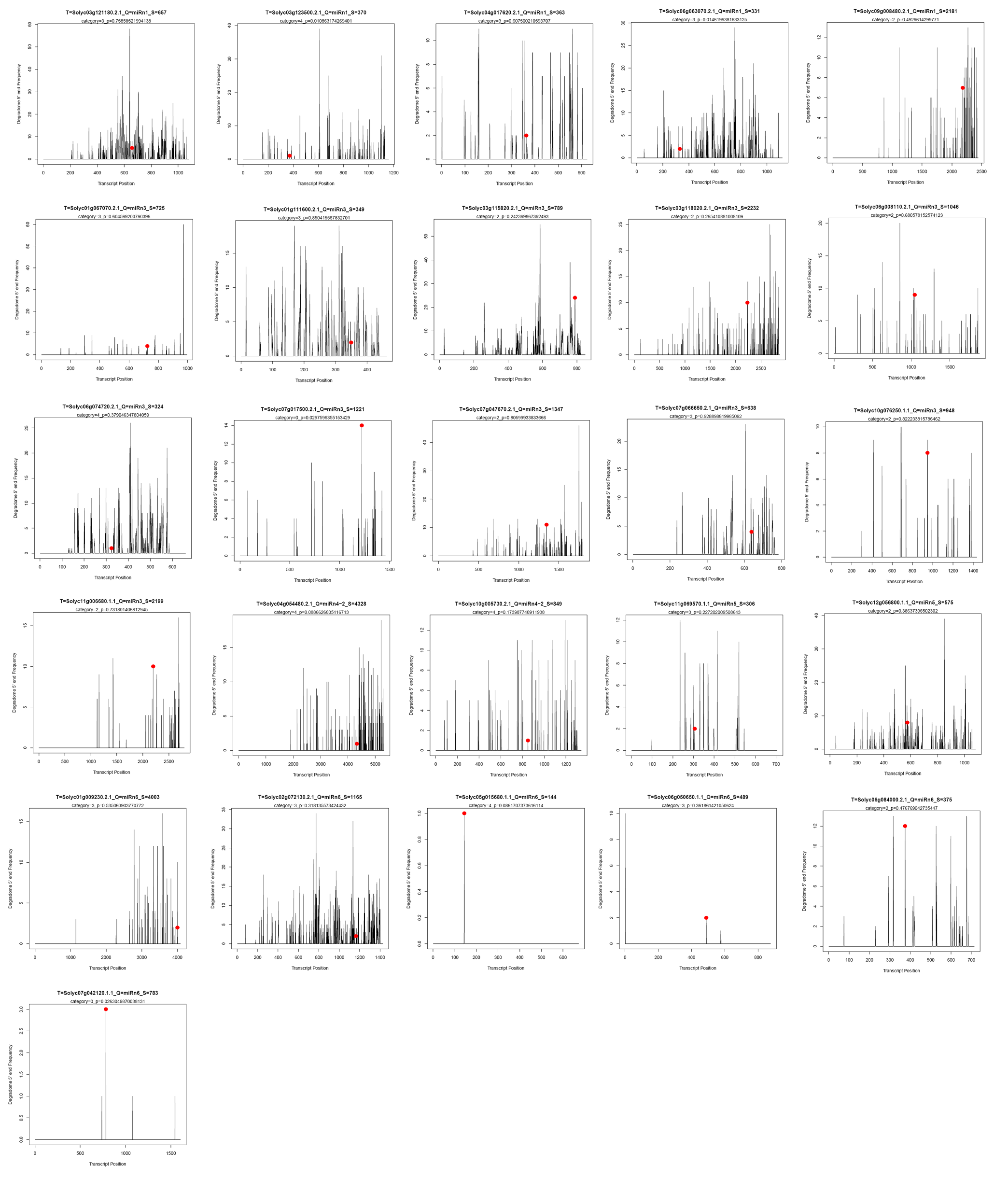

Supplement: Additional file 4: Figure S2. — Target plots (t-plots) of miRNAs targets confirmed by using degradome sequencing in tomato. [file 12870_2014_410_MOESM4_ESM.tiff]
