# Supplementary material for: Characterization of miRNAs associated with Botrytis cinerea infection of tomato leaves
Source: BMC Plant Biol. 2015 Jan 16;15:1. doi: 10.1186/s12870-014-0410-4 (PMC4311480; doi:10.1186/s12870-014-0410-4)
Supplement: Additional file 7: Table S5. — Primers used in this study. [file 12870_2014_410_MOESM7_ESM.doc]

Table S5 Primers used in this study.

| Target | Sequence | |
| --- | --- | --- |
| Probs of novel miRNA for gel blot analysis | | |
| miRn1_Prob | GACAACGAACTTGGGGTAAAT | |
| miRn3_Prob | AAAGCTCCTGACATATGCAAA | |
| miRn4_Prob | CCGTGATGCTCACTCAGCCAA | |
| miRn5_Prob | GTTGAGTAAGCACCAGAGAAAT | |
| miRn6_Prob | AATCCCCTACCATATTGCCAA | |
| miRn7_Prob | AGTCTAGACCTACATTGCTCA | |
| miRn8_Prob | CTTCCATAATCCTGTCACAGA | |
| miRNA primers for quantitative RT-PCR | | |
| miR156 | TTGACAGAAGATAGAGAGCAC | |
| miR159 | TTTGGATTGAAGGGAGCTCTA | |
| miR160 | TGCCTGGCTCCCTGTATGCCA | |
| miR169 | TAGCCAAGGATGACTTGCCTG | |
| miR319 | TTGGACTGAAGGGAGCTCCCT | |
| miR394 | TTGGCATTCTGTCCACCTCC | |
| miR1919 | ACGAGAGTCATCTGTGACAGG | |
| miR5300 | TCCCCAGTCCAGGCATTCCAAC | |
| miRn1 | ATTTACCCCAAGTTCGTTGTC | |
| U6-F | GGGGACATCCGATAAAATT | |
| U6-R | TGTGCGTGTCATCCTTGC | |
| Target mRNAs primers for RLM-RACE | | |
| RA44 | CGACUGGAGCACGAGGACACUGACAUGGACUGAAGGAGUAGAAA | |
| RA44OP | CGACTGGAGCACGAGGACACTGA | |
| RA44IP | GGACACTGACATGGACTGAAGGAGTA | |
| Solyc03g123500.2.1 | GSP1 | TCATCCTCCACGCTGTTCAT |
|  | GSP2 | TTACCTTAGCCTTCTTGCCTCT |
| Solyc06g063070.2.1 | GSP1 | AACTTCCGACAGAACAGATGAG |
|  | GSP2 | CTGCTTGGTTGCTGGTTTCT |
| Solyc03g115820.2.1 | GSP1 | AACTGCTGCAGGCCTTTTGC |
|  | GSP2 | CTTTTGCTTGTCTTGATCCCT |
| Solyc07g017500.2.1 | GSP1 | GGCTGCATATAAGGGTTCCC |
|  | GSP2 | GAGATTAGTATCCATCCGTGTTGT |
| Solyc04g054480.2.1 | GSP1 | GAAACCGAGGGGGTCCAGAT |
|  | GSP2 | TCAGTAGCCCTTAGACGGGG |
| Solyc10g005730.2.1 | GSP1 | CCTGCTCGTAGCCATTTTTTA |
|  | GSP2 | TCCGCTCAAAGGTGAAACAA |
| Solyc11g069570.1.1 | GSP1 | AGTTCGTTGGCTGTAGGGGC |
|  | GSP2 | TCCGAGTTGAGACCACGCTA |
| Solyc12g056800.1.1 | GSP1 | CCATCCAGTTACGAGTTCTTTG |
|  | GSP2 | GCAATGAAAAGTTCCCACCT |
| Solyc01g009230.2.1 | GSP1 | CAGATCAAGCAAAGTTTCCAAG |
|  | GSP2 | CATAATTCAGGCCACACTGTGT |
| Solyc06g050650.1.1 | GSP1 | CACGGTCCAGGAATATGTTGTA |
|  | GSP2 | AACACCACTCAGGCAATCCA |
| Target mRNA primers for quantitative RT-PCR | | |
| Solyc01g009070.2.1-F | AGAGCCCACATGGGCAATG | |
| Solyc01g009070.2.1-R | TCCAGCTGGAGGGGATTGA | |
| Solyc06g073640.2.1-F | CTGCCATCAACATCAGGATG | |
| Solyc06g073640.2.1-R | GAGAAGGAAGAGGGGAAGTAAC | |
| Solyc12g014120.1.1-F | TGGTGTTGTTTTCGGGGCTAA | |
| Solyc12g014120.1.1-R | CTCTGGAGTGAAGGGATATGGTC | |
| Solyc09g007810.2.1-F | GCCCCCACGAAAGAAACTAAG | |
| Solyc09g007810.2.1-R | ACCAACCGGAAACAAGCTAGA | |
| Solyc11g069500.1.1-F | AGCAGTCCTTTCTGTTGTTTATC | |
| Solyc11g069500.1.1-R | GTGGTAACAGTCCTGACGGTAAT | |
| Solyc11g013470.1.1-F | TGGACTCAGTAGCCCATCATTG | |
| Solyc11g013470.1.1-R | AACCCAAGGTTATTGCTCTTAACAT | |
| Solyc08g048370.2.1-F | ACCAAAACACACCGAGACAATGG | |
| Solyc08g048370.2.1-R | CCGTACCTCTGATAACGAGGCAAA | |
| Solyc08g048390.1.1-F | ATTACGGGTGAGCATAGAGCG | |
| Solyc08g048390.1.1-R | GCAAAGTGGAGGAAGAATTGG | |
| Solyc05g015520.2.1-F | CATTATCCCTGCCCCTCTACATT | |
| Solyc05g015520.2.1-R | TTACCCAAGCAAGTCATTCGAAC | |
| Solyc03g123500.2.1-F | AAGCCATTTGCCTTCTCTGCTT | |
| Solyc03g123500.2.1-R | CCTTTCCTTGGGTCACGTATTTC | |
| Actin-F | TGTGTTGGACTCTGGTGATGGTGT | |
| Actin-R | ATCCAAACGAAGAATGGCATGCGG | |
